# Genomic Sequences of Five *Helicoverpa armigera* Nucleopolyhedrovirus Genotypes from Spain That Differ in Their Insecticidal Properties

**DOI:** 10.1128/genomeA.00548-15

**Published:** 2015-06-11

**Authors:** Maite Arrizubieta, Oihane Simón, Trevor Williams, Primitivo Caballero

**Affiliations:** a Bioinsecticidas Microbianos, Instituto de Agrobiotecnología, UPNA-CSIC, Gobierno de Navarra, Mutilva, Spain; b Instituto de Ecología AC, Xalapa, Veracruz, Mexico; c Departamento de Producción Agraria, Laboratorio de Entomología Agrícola y Patología de Insectos, Universidad Pública de Navarra, Pamplona, Navarra, Spain

## Abstract

*Helicoverpa armigera* nucleopolyhedrovirus (HearNPV) has proved effective as the basis for various biological insecticides. Complete genome sequences of five Spanish HearNPV genotypes differed principally in the homologous regions (*hrs*) and the baculovirus repeat open reading frame (*bro*) genes, suggesting that they may be involved in the phenotypic differences observed among genotypes.

## GENOME ANNOUNCEMENT

The *Helicoverpa armigera* nucleopolyhedrovirus, HearNPV (*Baculoviridae*), is a single nucleocapsid alphabaculovirus that has proved to be an effective insecticide for control of *H. armigera* (Lepidoptera: Noctuidae) larvae on crops (1, 2). Five naturally occurring HearNPV isolates from Spain (3), named HearSNPV-SP1A, HearSNPV-SP1B, HearSNPV-LB1, HearSNPV-LB3, and HearSNPV-LB6, showed clear variation in insecticidal traits, such as occlusion body (OB) pathogenicity, speed of kill, and OB production in infected insects (3, 4). The genome of each of these genotypes was determined using the PacBio technique (Pacific Bioscience, USA) and assembled using the HGAP version 2.0.2 program.

The genome size of Spanish genotypes varied from 130,949 to 132,481 bp, with a G+C content of 39%, similar to that of other genotypes of HearSNPV (5–9). Overall, 136 open reading frames (ORFs) were present in HearSNPV-SP1A and HearSNPV-SP1B, whereas HearSNPV-LB1, HearSNPV-LB3, and HearSNPV-LB6 genomes lacked one ORF, as only two instead of three *bro* genes were located between homologous regions (*hrs*) 2 and 3. These 136 ORFs are present in at least one of the HearNPVs and *Helicoverpa zeae* SNPV genomes that have been sequenced previously (5–9). The 62 core genes (10) and 5 *hrs* were present in the Spanish genotypes, whereas only 10 of the 17 ORFs that are unique to *Helicoverpa* sp. SNPVs (7) were present in the five Spanish genotypes. ORF5 of unknown function was present in all the sequenced *Helicoverpa* sp. SNPVs (5–9). ORF58 (*bro-a*), unique to Chinese genotypes (5, 6), was not identified in the Spanish genotypes.

Although the insecticidal properties of the five Spanish genotypes showed significant differences between them, they had 98 to 99% nucleotide homology, with 43 ORFs that were 100% homologous among these genotypes. Compared to previously reported genotypes, Spanish variants had 95 to 99% nucleotide homology, and 13 ORFs presented 100% homology to HearNPV genotypes registered in GenBank (5–9).

The principal variation among the Spanish genotypes was located in the homologous regions (*hrs*) and baculovirus repeat ORFs (*bro*) genes, suggesting possible involvement in genotype-specific variation in insecticidal traits. Phylogenetic analysis indicated that the genotype from Kenya (HearNPV-NNg1) was more similar to Spanish genotypes than variants from China (HearNPV-C1, HearNPV-G4) or, Australia (HearNPV-Au) or *Helicoverpa zeae* SNPV from the United States, possibly reflecting biogeographical relationships among populations of *H. armigera*, which has its origin in Africa. The complete genome sequences presented in this study provide a useful resource for further exploring the genetic diversity and the factors that determine the insecticidal properties of alphabaculoviruses.

### Nucleotide sequence accession numbers.

HearSNPV-LB1, HearSNPV-LB3, HearSNPV-LB6, HearSNPV-SP1A, and HearSNPV-SP1B genome sequences were submitted to GenBank with the accession numbers KJ701029, KJ701030, KJ701031, KJ701032, and KJ701033, respectively.
